# GSK3β and ERK regulate the expression of 78 kDa SG2NA and ectopic modulation of its level affects phases of cell cycle

**DOI:** 10.1038/s41598-017-08085-9

**Published:** 2017-08-08

**Authors:** Shweta Pandey, Indrani Talukdar, Buddhi P. Jain, Goutam K. Tanti, Shyamal K. Goswami

**Affiliations:** 1 School of Life Sciences, Jawaharlal Nehru University, 110067 New Delhi, India; 2 Department of Zoology, School of Life Sciences, Mahatma Gandhi Central University, Motihari, 845401 Bihar India; 3 Department of Neurology, School of Medicine, Technical University of Munich, Munich, Germany

## Abstract

Striatin and SG2NA are essential constituents of the multi-protein STRIPAK assembly harbouring protein phosphatase PP2A and several kinases. SG2NA has several isoforms generated by mRNA splicing and editing. While the expression of striatin is largely restricted to the striatum in brain, that of SG2NAs is ubiquitous. In NIH3T3 cells, only the 78 kDa isoform is expressed. When cells enter into the S phase, the level of SG2NA increases; reaches maximum at the G2/M phase and declines thereafter. Downregulation of SG2NA extends G1 phase and its overexpression extends G2. Ectopic expression of the 35 kDa has no effects on the cell cycle. Relative abundance of phospho-SG2NA is high in the microsome and cytosol and the nucleus but low in the mitochondria. Okadoic acid, an inhibitor of PP2A, increases the level of SG2NA which is further enhanced upon inhibition of proteasomal activity. Phospho-SG2NA is thus more stable than the dephosphorylated form. Inhibition of GSK3β by LiCl reduces its level, but the inhibition of ERK by PD98059 increases it. Thus, ERK decreases the level of phospho-SG2NA by inhibiting GSK3β. In cells depleted from SG2NA by shRNA, the levels of pGSK3β and pERK are reduced, suggesting that these kinases and SG2NA regulate each other’s expression.

## Introduction

Striatin, S/G2 nuclear autoantigen (SG2NA), and zinedin constitute a three-member subfamily of WD-40 repeat protein superfamily. Apart from WD-40 repeats, they have a caveolin-binding motif, a coiled-coil structure, and a calmodulin-binding domain^1,2^. They also share a number of smaller motifs, suggesting conservation of function(s)^3^. In agreement, supramolecular signalling complex(s) named STRIPAK (Striatin-interacting phosphatases and kinases) assembled around striatin containing both kinases and phosphatases have been described^4–7^. Recent studies suggest that STRIPAK complexes regulate several nodal signalling pathways involved in cell proliferation, differentiation, polarity, apoptosis and metabolism^7,8^. Impairment in its function has been linked with diseases like autism, cancer, diabetes, cerebral cavernous malformation etc.^6,7^.

SG2NA was first characterised as an auto**a**ntigen from a cancer patient. Subsequent studies suggested that it is a **n**uclear protein/**a**ntigen with increased expression during **S** and **G2** phases of cell cycle and named accordingly^9^. It has at least six isoforms generated by alternative splicing and RNA editing^10,11^. These variants are differentially expressed in mouse tissues and cultured cells^11^. Variants of SG2NA have similar but distinctive structural characteristics and are likely to have related functions^12^. Because of extensive conservation of various sequence motifs, variants of SG2NA are expected to have overlapping but distinct function. Down regulation of SG2NA by shRNA makes Neuro2A cells more susceptible to oxidative stress but specific contribution by each variant is yet to be determined^13^.

Cell cycle is the key requirement for propagation and sustenance of all organisms. It is tightly controlled, ensuring incidence of correct event in a highly conserved and orchestrated manner^14,15^. Cyclin dependent kinases in association with corresponding cyclins create an intricate network of phosphorylation and dephosphorylation of respective targets ensuring the fidelity of cell cycle progression^16,17^. It has thus become increasingly important to understand how these proteins interact and coordinate with each other ensuring a flawless network of events.

Till date, a wide range of cellular events have been linked to striatin and SG2NA. They act as a subtype of B subunit of serine/threonine phosphatase, protein phosphatases 2 A (PP2A)^18^, determining its specificity and subcellular localization. PP2A counteracts CDK-dependent phosphorylation of cell cycle proteins throughout cell division^19^. Other interacting partners of SG2NA are cortactin binding protein 2 (CTTNBP2) (involved in microtubule stability and dendritic spinogenesis)^20^, Mob3 (involved in membrane trafficking)^18^, APC (regulate tight junctions)^21^, chaperonin containing TCP-1/TCP-1 ring complex (CCT/TRiC), a chaperonin^4^, Gαi and ERα^22^.

Although SG2NA was initially characterized as a cell cycle regulated protein^9^, its precise function in this context has not been explored. Here, in this manuscript, we have studied the role of SG2NA on cell cycle progression. We demonstrate that the level of SG2NA is modulated during cell cycle while over- or under expression of SG2NA alters the duration of phases. Also, the stability of SG2NA is regulated by its phosphorylation by glycogen synthease kinase 3β (GSK3β) and extracellular signal regulated kinases (ERK), while SG2NA in turn controls the level of these kinases. Therefore, a precisely controlled feedback-feedforward mechanism integrating the kinase-phosphate signalling involving SG2NA regulates certain aspects of cell cycle progression.

## Results

### 78 kDa SG2NA is the prevailing isoforms in NIH3T3 cells

**Figure 1:**
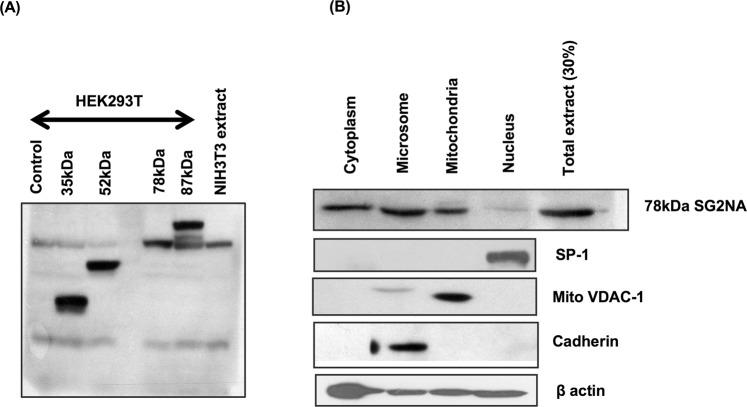
NIH3T3 has only 78 kDa isoform enriched in cytosol and microsomes. (**A**) NIH3T3 cells were harvested and 75 μg of protein lysates were loaded on SDS-polyacrylamide gel and blotted with SG2NA antibody. Protein was extracted from transiently transfected HEK293T cells harbouring various isoforms of SG2NA i.e., 35 kDa, 52 kDa, 78 kDa and 87 kDa were loaded to act as markers. (**B**) NIH3T3 cells were fractionated and each organelle rich fraction was loaded and blotted with SG2NA antibody, authenticity of the fractions were confirmed by probing with organelle specific markers. Full length blots for panel (B) are shown in Supplementary Figure S1.

We have observed that NIH3T3 cells primarily express 78 kDa isoform of SG2NA (Fig. 1A). However, it is the most abundant but not exclusive isoform in different tissues^11^. Owing to the prevalence of only one isoform, we selected NIH3T3 cells as a model for studying the role of SG2NA in cell physiology. Though SG2NA was initially reported as nuclear antigen, later studies have shown that it is localized in multiple cellular compartments including mitochondria, cytosol and plasma membrane^10,23^. We reiterated the subcellular distribution of 78 kDa SG2NA in NIH3T3 cells. Cells were fractionated by osmotic alteration followed by homogenisation in Dounce homogeniser and differential centrifugation. The enriched fractions were then subjected to western analysis along with organelle specific markers. As showed in Fig. 1B, 78 kDa isoform was most abundant in cytosol, followed by microsomes and mitochondria, while nucleus had a barely detectable presence.

### SG2NA is modulated during progression of cell cycle

**Figure 2:**
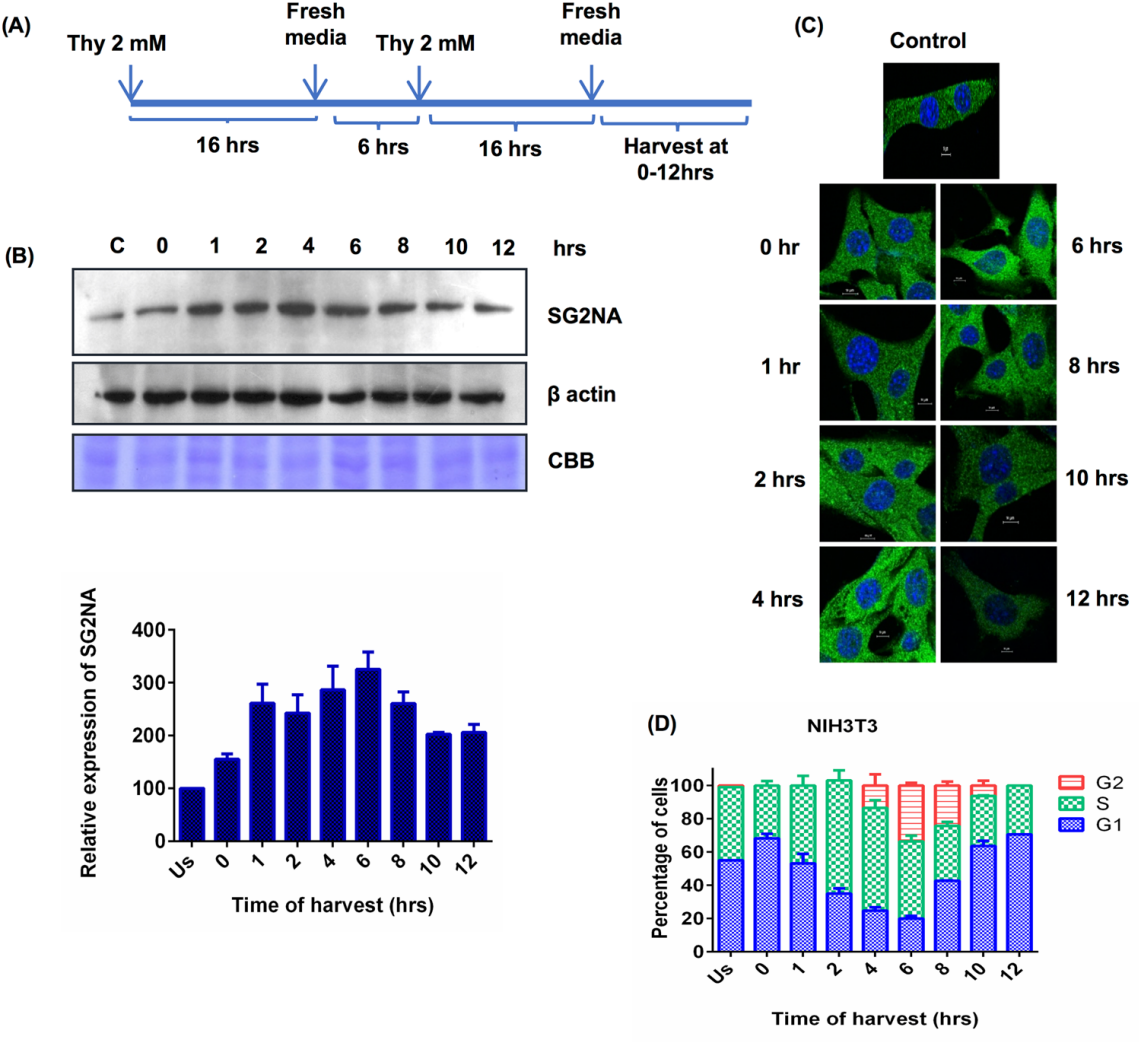
SG2NA is modulated during the course of cell cycle. (**A**) Schematic representation of synchronisation of cells using double thymidine block. (**B**) *Upper panel*, NIH3T3 cells were synchronised and allowed to enter the cell cycle, cells were harvested at different time points and 75 μg protein lysates were loaded on SDS-polyacrylamide gel followed by probing with SG2NA and β-actin antibodies. Equal loading of protein was shown by CBB stained gel. *Bottom panel*, Graph representing quantitation of western analysis of SG2NA level during the course of cell cycle. (**C**) Confocal images of synchronised NIH3T3 cells at various time points of cell cycle. Magnification- 60X, scale bar - 10 μm. (**D**) Graph representing percentage of cells present in various phases of cell cycle at different time of harvest. Error bars are the standard deviation of 3 experiments. Full length blots for panel (B) are shown in Supplementary Figure S1.

SG2NA was initially reported to be cell cycle regulated protein whose expression is augmented during the S to G2 phases and named accordingly^24^. However, its mechanistic role in cell cycle progression has not been investigated. Subsequent identification of multiple variants by us further necessitated to have an insight into role of each isoform, if any, in cell proliferation. Since NIH3T3 cells express only one variant, we considered it better suited to address this question. Cells were synchronised using double thymidine block and then allowed to enter the cell cycle (Fig. 2A). Cells were harvested at different time points and level of SG2NA was analyzed by western blotting. As shown in Fig. 2B, expression of SG2NA gradually increased as cells enter the cycle, reached maximum at 6 hrs and declined thereafter. Confocal images of synchronised cells immunostained with SG2NA antibody corroborated the western data (Fig. 2C). Simultaneous FACS analysis (Fig. 2D) suggested that its expression increased as the cells enter S phase, reaches maximum towards G2/M phase, followed by a decline.

### Down regulation SG2NA increases the duration of G1 phase while its upregulation increases that of the G2 phase

**Figure 3:**
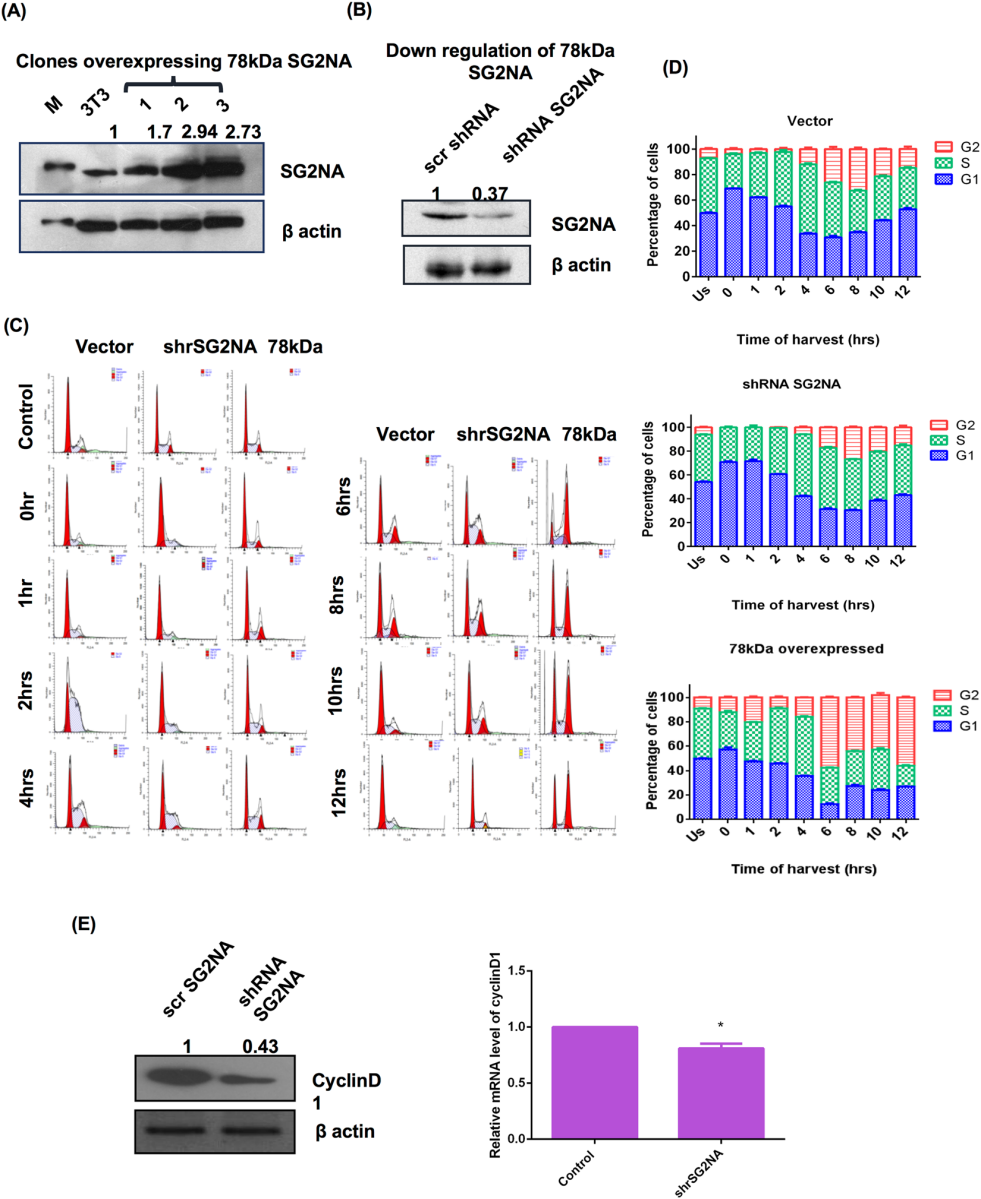
SG2NA downregulation increases the G1 phase duration while upregulation increases G2 phase duration. (**A**) Cells stably overexpressing 78 kDa and (**B**) underexpressing SG2NA were harvested, 100 μg of cell lysates were loaded on SDS-polyacrylamide gel and immunoblotted with SG2NA and β-actin antibody. Numbers on the top of bands represent fold change in protein levels relative to untransfected NIH3T3 cells. (**C**) Representative FACS histograms of NIH3T3 cells stably transfected with vector alone, 78 kDa SG2NA and shRNA against SG2NA. (**D**) Graphical representation of the percentage of cells present in different phases of cell cycle during the cell cycle preogression. (**E**) *Left panel:* Lysates of normal NIH3T3 cells and SG2NA downregulated cells were loaded on SDS-polyacrylamide gel and probed with cyclin D1 and β-actin. *Right panel*: Graph representing real time analysis by qPCR for cyclin D1 mRNA expression in normal NIH3T3 cells and SG2NA downregulated cells. Error bars are the standard deviation of 3 experiments. *P < 0.05, **P < 0.01, ***P < 0.001. Full length blots for panel (A) and (B) are shown in Supplementary Figure S1.

To delineate role of SG2NA in cell cycle, cells over- and under expressing SG2NA (78 kDa) were developed. Upregulation of its expression was achieved by stably transfecting the recombinant *myc* tagged cDNA under a constitutive promoter and it was down regulated by the stable expression of an shRNA specifically targeting it^23^. Differential expression of the respective proteins was confirmed by western blot (Fig. 3A and B). Cells were synchronised using double thymidine block and FACS analysis was done to track the cell cycle progression. As shown in Fig. 3C, cells with downregulated SG2NA were confined to the G1 phase for a longer period of time (~42% ± 0.545 of cells were at G1 at 4 hrs) as compared to the cells expressing vector only (~33% ± 0.409 cells were at G1 at 4 hrs). Similarly, in those cells, at the time of release (from double thymidine block), more cells (~54.107% ± 0.47) were at G1 phase as compared to the normal counterpart (~49.793% ± 0.547). In contrast, cells overexpressing 78 kDa had elongated G2 phase that lasted beyond 12 hrs (of release from thymidine block) as compared to control which complete G2 phase and entered next G1 phase by 10 hrs. Figure 3D, represents the relative distribution of phases of cell cycle in normal 3T3 cells, 78 kDa overexpressing cells and cells with reduced expression of SG2NA.

It has been known that downregulation of cyclin D1 leads to G1 cell cycle arrest and GSK3β is responsible for its phosphorylation and degradation^25,26^. We thus estimated cyclin D1 level in SG2NA knockdown background. As shown in Fig. 3E, down regulation of SG2NA led to reduction of cyclin D1 mRNA and protein, suggesting a mechanistic association between reduced level of SG2NA, cyclin D1 level and elongation of G1 phase.

### Phosphorylation of SG2NA oscillates during the progression of cell cycle

**Figure 4:**
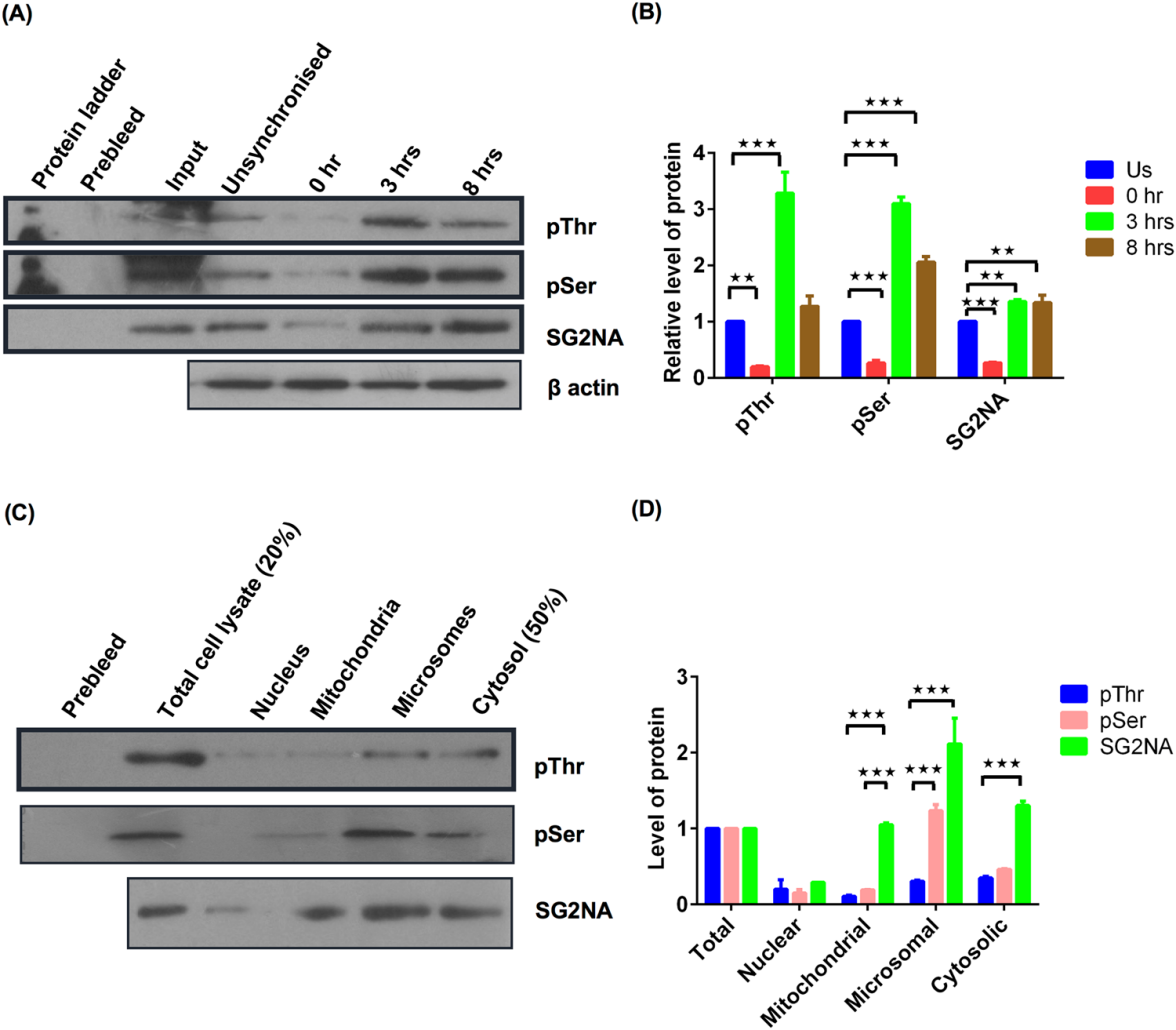
Phosphorylation of SG2NA is oscillated during the course of cell cycle and is organelle specific. (**A**) NIH3T3 cells were synchronised by double thymidine block and harvested at different phases as G1, S, G2 at 0 hr, 3 hrs and 8 hrs after release from thymidine block. Then cell lysates were immunoprecipitated with SG2NA antibody followed by probing using phosphoserine, phosphothreonine, SG2NA and β-actin antibodies. (**B**) Graph representing relative level of proteins with respect to β-actin as a control. (**C**) NIH3T3 cells were fractionated and organelle enriched fractions were immunoprecipited with SG2NA antibody and then probed with phosphoserine, phosphothreonine and SG2NA antibodies. (**D**) Graph representing level of proteins in various organelle riched fractions. Error bars are the standard deviation of 2 independent experiments. *P < 0.05, **P < 0.01, ***P < 0.001. Full length blots for panel (A) and (C) are shown in Supplementary Figure S1.

Since cell cycle regulation is largely controlled by phosphorylation-dephosphorylation of different regulatory proteins, we also looked into the phopshorylation status of SG2NA over progression of cell division. NIH3T3 cells were synchronised by double thymidine block and harvested at different time points with the progression of cell cycle. Cell lysates were immunoprecipitated with the SG2NA antibody, followed by probing with phospho-serine and phospho-threonine antibodies. As shown in Fig. 4A, the level of phosphorylated SG2NA was low at G1 phase but augmented during S and G2 phase. SG2NA is primarily localized at cytoplasm, microsome and mitochondria (Fig. 1B). We thus asked whether phosphorylated SG2NA maintains same subcellular profile. To answer this, cells were fractionated and each organelle rich fraction was subjected to immunoprecipitation by SG2NA antibody followed by western blotting using phospho-serine and phospho-threonine antibodies. We observed that mitochondrial SG2NA was less phophorylated while nuclear (least abundant), microsomal and cytosolic SG2NAs were more phosphorylated (Fig. 4C). Figure 4B and D represent the graph showing relative expression of the proteins in different time points and various organelle riched fractions respectively.

### The hyperphosphorylated form of SG2NA is more stable and its level is regulated by proteasomal degradation

**Figure 5:**
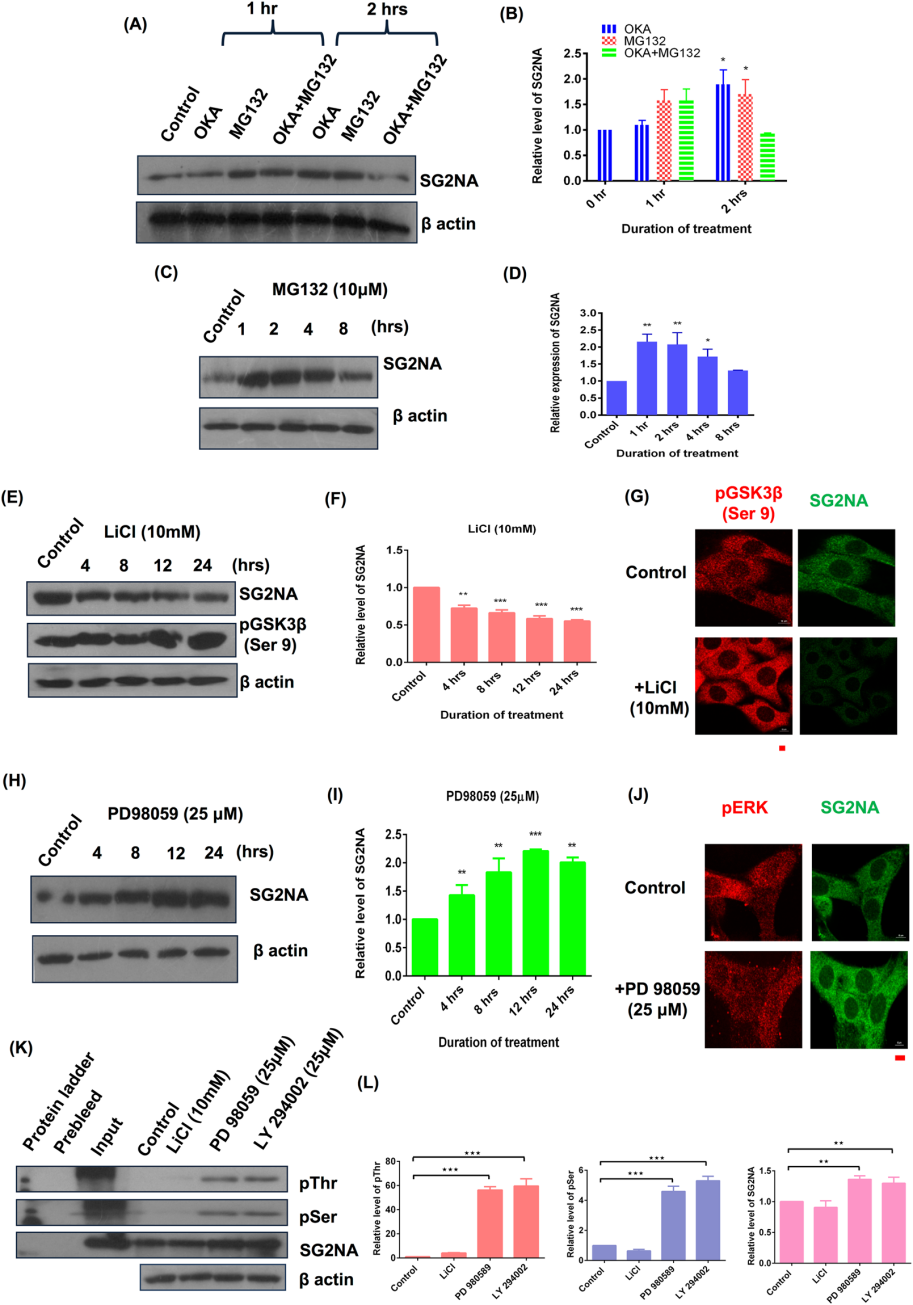
SG2NA is regulated at protein level by proteasomal degradation and its hyperphosphorylated form is more stable. NIH3T3 cells were treated with (**A**) 100 μM Okadoic acid alone and 10 μM MG132 and mixture of both (**C**) 10 μM MG132 alone (**E**) 10 mM LiCl (**H**) 25 μM PD98059, for various time points and western analysis was done with SG2NA and β-actin antibody. Graph representing quantitaive analysis of relative expression of SG2NA in time dependent manner upon their treatment is shown in (**B**), (**D**), (**F**) and (**I**) repectively. Confocal images of the cells treated with LiCl and PD98059 is shown in (**G**) and (**J**) respectively. Magnification- 60X, scale bar- 10 μm. (**K**) NIH3T3 cells were treated with various inhibitors as LiCl, PD98059 and LY294002 for 12 hrs and cell lysates were immunoprecipitated with SG2NA antibody followed by blotting with phosphoserine, phosphothreonine and SG2NA antibodies. Equal loading is shown by β-actin. (**L**) Graphs representing relative level of respective proteins with β-actin as loading control is shown. Error bars are the standard deviation of 2 independent experiments. *P < 0.05, **P < 0.01, ***P < 0.001. Full length blots for panel (A), (C), (E), (H) and (K) are shown in Supplementary Figure S1.

SG2NA and striatin have been associated with the serine/threonine phosphatase PP2A which in turn regulates several kinases^27,28^. However, whether these kinases/phosphatases also regulate the expression of SG2NA has not been explored. We first examined whether SG2NA is part of an autoregulatory loop involving its dephosphorylation by PP2A. Cells were treated with okadoic acid (100 nM), an inhibitor of PP2A and proteasomal inhibitor MG132. As shown in Fig. 5A, treatment with okadoic acid for 1 hr had no effect on the level of SG2NA but at 2 hrs, there was about two fold increase in its level. Interestingly, treatment with MG132 (10 μM) either alone or along with okadoic acid also resulted in an increased SG2NA level as compared to the respective controls (Fig. 5C). This suggests that hyperphosphorylated form of SG2NA is more stable than the phosphorylated form. Moreno *et al*., (2000), have reported that SG2NA remains phophorylated under normal condition and therefore, it might be a dynamically regulated by continuous degradation and replenishment. To test that, we treated cells with MG132 for various durations and observed that its level gradually increased till 2 hrs, followed by a decrease till 8 hrs, the last time point tested. In order to find out the kinase responsible for the phosphorylation of SG2NA, cells were treated with LiCl (10 mM), the inhibitor of GSK3β which resulted in a sharp decrease in the level of SG2NA (Fig. 5E). Confocal images of cells treated with LiCl and PD98059 further support the western data (Fig. 5G,J). Therefore, GSK3β maintains the phosphorylated state of SG2NA, enhancing its stability, while its inhibition by LiCl results in dephosphorylated SG2NA that is degraded. On the contrary, when cells were treated with the ERK inhibitor PD98059, there was an increase in SG2NA level by several folds (Fig. 5H). Graph representing quantitation of relative level of SG2NA with duration of these treatments has been shown in Fig. 5B,D,F and I. Such reciprocal effect of GSK3β and ERK on the stability of SG2NA can be explained by the interrelationship of these two kinases. GSK3β is phosphorylated by ERK and Akt thereby inhibiting its activity. To confirm this interrelationship, we treated NIH3T3 cells with inhibitors of ERK (PD98059), GSK3β (LiCl), and Akt (LY294002); immunoprecipitated the extracts with SG2NA antibody and probed it with phospho-serine/threonine antibody. As expected, level of phosphorylated SG2NA decreased when cells were treated with GSK3β inhibitor LiCl but it increased when treated with inhibitors of ERK and Akt (Fig. 5K). The quantitation of the data is shown in the graph (Fig. 5L).

### SG2NA also regulates the level of pERK and pGSK3β

**Figure 6:**
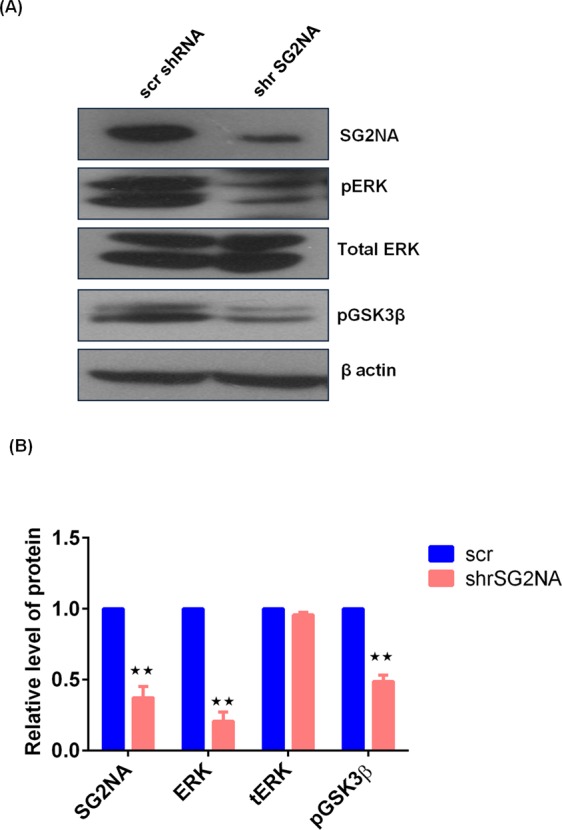
SG2NA also regulates the level of pERK and pGSK3β. (**A**) NIH3T3 and SG2NA downregulated NIH3T3 cells were harvested and loaded on SDS-polyacrylamide gel, transferred then probed with various antibodies like SG2NA, pERK, total ERK, pGSK3β and β-actin. (**B**) Graph representing relative expression of proteins with β-actin as loading control is shown. Error bars are the standard deviation of 2 independent experiments. *P < 0.05, **P < 0.01, ***P < 0.001. Full length blots for panel (A) are shown in Supplementary Figure S1.

We have reported earlier that downregulation of SG2NA results in the reduction of pAkt level^23^. We thus analysed the expression of other two kinases i.e., GSK3β and ERK in SG2NA knockdown background. As shown in Fig. 6A, in SG2NA knockdown background, expression levels of pGSK3β and pERK were also low, suggesting a feedback control mechanism regulating the expression of SG2NA vis-a-vis these kinases. Figure 6B represents the quantitative analysis of the above data.

### 35 kDa variant of SG2NA has no role in cell cycle

**Figure 7:**
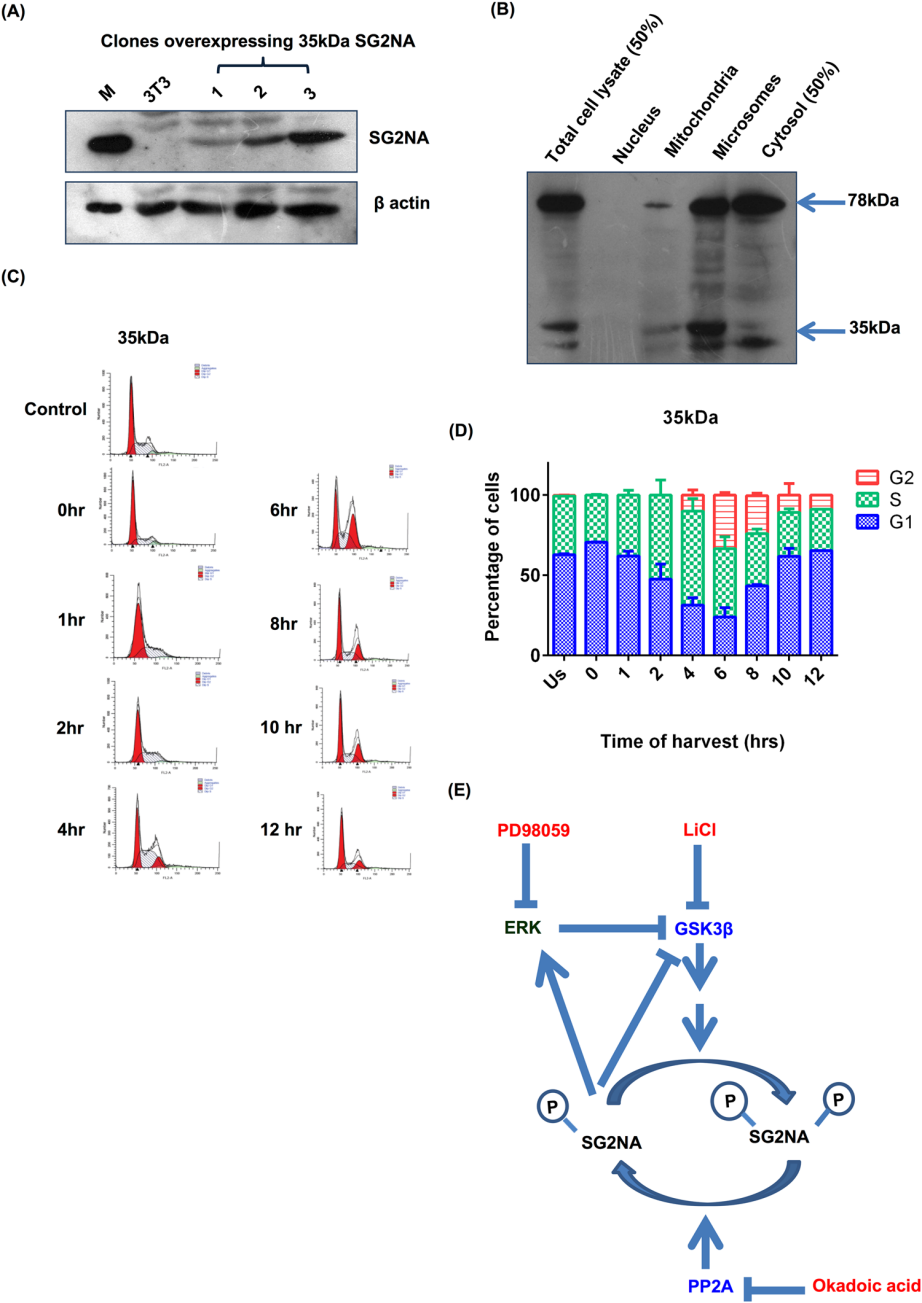
35 kDa variant of SG2NA has no role in cell cycle. (**A**) Cells stably overexpressing 35 kDa were harvested, 100 μg of total protein lysate was loaded on SDS-polyacrylamide gel and probed with SG2NA and β-actin. (**B**) 35 kDa overexpressing NIH3T3 cells were fractionated and organelle rich fractions loaded on SDS-polyacrlamide gel followed by western analysis using SG2NA antibody. (**C**) Representative FACS histograms of NIH3T3 cells stably transfected with 35 kDa SG2NA. (**D**) Graphical representation of the percentage of cells present in different phases of cell cycle during the cell cycle preogression. Error bars are the standard deviation of 3 independent experiments. (**E**) Schematic representation of proposed process. SG2NA remains in hyperphosphorylated state in the presence of okadoic acid. This hyperphosphorylated SG2NA is more stable. PD98059 inhibits phosphorylation of MAPK kinase and thus ERK. The decrease in phosphorylation of ERK results in decreased activity of ERK thereby decreasing inhibitory phosphorylation of GSK3β which inturn increases pSG2NA level thus increases its stability. LiCl is known to inhibit GSK3β activity by increasing its Ser-9 phosphorylation, thereby not phosphorylating SG2NA hence decreased level of SG2NA was observed. Full length blots for panel (A) are shown in Supplementary Figure S1.

35 kDa variant of SG2NA is devoid of the C-terminal WD40 repeats, a domain often involved in interaction with other proteins. To test if it has any role in cell cycle, it was overexpressed (*myc* tagged cDNA) in NIH3T3 cells to see if it might have any dominant negative effect on its full-length counterpart i.e., 78 kDa isoform (Fig. 7A). Like 78 kDa variant, 35 kDa was also localized in cytosol, microsomes and mitochondria with a barely detectable level in nucleus (Fig. 7B) although it was more abundant at microsomal fraction. These cells didn’t show any significant change in cell cycle pattern (Fig. 7C and D). This indicates that 35 kDa SG2NA might have some other functions in cell physiology.

## Discussion

Multiple proteins with partially overlapping sequences are generated from a single gene by alternative splicing. Generally, such splice variants have similar structures with non-redundant but analogous functions. Our protein of interest SG2NA has six such variants of which only one is expressed in NIH3T3 cells (Fig. 1 shown above). Previously we have reported that three other cell lines viz., Neuro2A, CHO and C6 cells also express 78 kDa as the prevalent form, while only C6 cells has a very low level of expression of 52 kDa variant^23^. In contrast, mouse tissues shows higher levels of expression of multiple isoforms like 78, 52, 35 and 87 kDas, suggesting that other forms of SG2NA might have cell type or context specific functions.

SG2NA was originally reported as a cell cycle related protein as its expression is augmented during S to G2 phases^9^. Later studies associated SG2NA and two other members of the subfamily i.e., striatin and zinedin; with multimeric signalling assembly named STRIPAK^7^. Whether the suggested role of SG2NA in cell cycle progression and STRIPAK assembly are interlinked, has not been examined. To address the function of 78 kDa form, we first re-examined its modulation during progression of cell cycle with a population of synchronized NIH3T3 cells. In agreement with the earlier report, its expression augmented during progression from S to G2 phases. This reiteration was necessary as another related observation that SG2NA is nuclear resident protein was found to be incorrect in our study^23^. Association of SG2NA with cell cycle is not merely circumstantial as its downregulation extended G1 phase while its overexpression extended G2 phase.

Propagation of any organism essentially requires a high fidelity of replication of the constituent cells. Multiple feed-forward and feedback mechanisms ensure such precise duplication of mother to daughter cells. The mechanism cell cycle is thus highly conserved in evolution from unicellular eukaryotes like yeasts to higher metazoans like humans. However, despite high level of conservation in its mechanisms, cell division process is much more complex in higher organisms as it is regulated by a plethora of signals like growth factors, cytokines and small metabolites. Over the past quarter of a century, numerous proteins have been associated with cell division, but among them, of paramount importance are the cyclins and dependent kinases^29^. In mammalian cells, upon mitogenic stimulation, CDK4/5 are activated in the G1 phase, allowing the cells to transverse the restriction point^30^. Although numerous CDK substrates have been described till date, except for a few, the detailed mechanisms by which CDK mediated phosphorylation controls their function is largely unknown. Another important arm of cell cycle regulation is the protein phosphatase PP2A mediated dephosphorylation of pocket proteins^31^. Since SG2NA is a part of the STRIPAK as well as the PP2A assemblies, partial loss of SG2NA likely impairs the kinase-phosphatase network, prolonging the G1 phase, a hallmark of cell cycle arrest. On the other hand, hyperactivity of SG2NA by it’s over expression might trip the balance between the feed-forward and feedback mechanisms controlled by kinases and phosphatases, prolonging the G2 phase. Such proposition is substantiated by our observation that downregulation of SG2NA leads to the decrease in CDK1 both at RNA and protein levels.

We have reported earlier that SG2NA interacts with a number of proteins including DJ-1, growth arrest specific protein 8, mitogen activated protein kinase 4 and death domain associated protein^23^. Although none of these proteins have an established role in cell cycle, they all have context specific regulatory functions. Further, these proteins were identified as interacting partners of SG2NA by an *in vitro* assay that is not necessarily all inclusive. So, if there are other cell cycle associated proteins interacting with SG2NA *in vivo*, they are yet to be identified. To circumvent this limitation and to have an insight into function of SG2NA in the G1 and G2 phases, we tested state of its phosphorylation during the progression of cell division. As anticipated, the level of phosphorylated SG2NA (at Serine/Threonine) was low at G1 phase but it increased during S and G2 phases, suggesting a differential function during the progression of the process. Interestingly, the extent of phosphorylation was lesser in mitochondria as compared to that in cytosolic, microsomal and nuclear fractions. Any interpretation of such differential phosphorylation of SG2NA at this stage might be empirical as role of these organelles in cell cycle regulation is just emerging^32–34^. ERK and GSK3β are the two signalling kinases that modulate a plethora of cellular events including cell proliferation^35,36^. ERK is generally considered to be a growth promoting kinase, although it has also been associated with growth arrest and apoptosis^35^. Downregulation of SG2NA delays the G1 phase, a sign of growth arrest. It is thus likely to have growth promoting functions, which is also corroborated by the fact that its overexpression extends G2 phase. On the contrary, inhibition of ERK stabilizes SG2NA through phosphorylation, suggesting a growth inhibitory role. We addressed this dichotomy by showing that ERK is linked to SG2NA via GSK3β. We propose that ERK phosphorylates GSK3β, resulting in its degradation which in turn affects the phosphorylation of SG2NA. Dephosphorylated/ hypophosphorylated SG2NA is then degraded, disfavouring growth. Existence of such ERK-GSK3β-SG2NA axis is in full agreement with the earlier reports. Cyclin D1 senses the mitogenic signals and phosphorylates the retinoblastoma protein, facilitating the transition from G1 to S phase. During cell cycle, the rate of synthesis of cyclin D1 and its degradation is tightly regulated by ERK and GSK3β^37,38^. The X protein of hepatitis B virus (HBV-X) activates ERK which then phosphorylates GSK-3β, resulting in its inactivation and upregulation of β-catenin^39^. Integration of SG2NA to the ERK and GSK3β signalling is further evident from the observation that in SG2NA knockdown background, both pERK and GSK3β are down regulated. However, details investigation will be required in future to have a better understanding of this ERK-GSK3β and 78 kDa SG2NA axis. Another silent feature of our study is that ectopically expressed 35 kDa SG2NA does not affect the progression of cell cycle, despite its presence in mitochondria, microsomes and cytosol. Currently we are using tools of cellular and molecular biology to investigate the roles of this and other variants of SG2NA (52, 82 and 87 kDas) in cellular processes.

## Materials and Methods

### Reagents

All chemicals are procured from Sigma, USA unless mentioned otherwise. Plastic wares used in tissue culture were from Corning, Sigma–Aldrich, USA.

### Antibodies

Mouse monoclonal anti-β-actin (Sigma, #A1978, 1:5000 for WB); mouse monoclonal anti-myc (Cell Signaling Technology, #2276, 1:4000 for WB; 1:1000 for IF), goat polyclonal anti-VDAC (SantaCruz Biotechnology, #sc-8828, 1:1000 for WB), rabbit polyclonal histone H3 (AbCam, #ab1791), rabbit polyclonal cadherin antibody (NeoBioLab, #A1550, 1:3000 for WB), mouse monoclonal striatin3 antibody (S68) (Pierce, #MA1–46461, 1:3000 for WB; 1:5000 for IF and Santa Cruz Biotechnology, #sc-13562), phospho-GSK-3β (Ser9) antibody (CST, #9336), P-Thr-polyclonal rabbit antibody (CST, #9381), mouse monoclonal P-Ser antibody (Sigma,#P3430), mouse monoclonal anti-cyclinD1 (SantaCruz #sc20044).

### Cell culture

HEK293T and NIH3T3 cells were procured from Cell Repository, NCCS, Pune, India. The cells were cultured in DMEM containing 10% foetal bovine serum (FBS), 100 U/ml penicillin, 100 μg/ml streptomycin and maintained at 37 °C, 5% CO_2_ in humified atmosphere.

### Constructs

ORF of various isoforms of DNA were cloned in pcDNA3.1/myc-His(-)B from Clonetech.

### Cell lysis and immunoblotting

Cell lysates preparation and immunoblotting was performed as described earlier^23^, briefly cells were lysed in ice-cold buffer containing 50 mM Tris pH 7.6, 400 mM NaCl, 1 mM EDTA, 1 mM EGTA, 1% NP-40, 1 mM sodium orthovanadate, 10 mM sodium fluoride, protease inhibitor cocktail and 1 mM PMSF. Protein estimation was done using BCA method. Lysates were resolved on 10–12% polyacrylamide SDS gel followed by transfer to PVDF membrane and probed with respective primary and secondary antibodies. Signals were developed using ECL detection system.

### Developing stable cell lines

Plasmids were linearised and transfected in NIH3T3 cells. 72 hrs post transfection cells were subjected to selection pressure, i.e., 7 μg/ml puromycin, and after clonal selection, maintained at 3 μg/ml puromycin.

### Sub-cellular fractionation

Cells were harvested by centrifugation at 200 g for 7 mins. The pellet was resuspended in an isotonic solution containing 250 mM sucrose, 10 mM HEPES[4-(2-hydroxyethyl)−1-piperazineethanesulfonic acid.]-Tris pH7.4 and 1 mM ethyleneglycolbis(2-aminoethyl ether)tetraacetic acid(EGTA)-Tris and again centrifuged at 200 g for 7 mins. The pellet was resuspended in a hypotonic solution (2 ml) containing 100 mM sucrose, 10 mM HEPES-Tris and 1 mM EGTA and homogenised using Dounce homogeniser, followed by addition of hypertonic solution (1.96 ml) of 1.78 M sucrose, 10 mM HEPES-Tris pH 7.4, 1 mM EGTA-Tris and 2 mM MgCl_2_. The resulting isotonic solution was centrifuged at 200 g for 10 mins to obtain the nuclear pellet, followed by centrifugation at 300 g for 10 mins, 5000 g for 10 mins and 100,000 g for 1 hr to obtain post nuclear pellet, mitochondrial pellet and microsomal pellet respectively. The supernatant obtained was saved as cytosolic fraction. These fractions were further enriched using OptiPrep density gradient medium (60% iodixanol solution, Sigma, #D1556) as per manufacturer’s protocol.

### Immunoprecipitation

Cells were lysed in immunoprecipitation buffer containing 50 mM Tris, pH 7.6, 400 mM NaCl, 1 mM ethylenediaminetetraacetic acid (EDTA), 1 mM EGTA, 1% NP-40, 1 mM sodium orthovanadate, 10 mM sodium fluoride, protease inhibitors and 1mM PMSF on ice followed by centrifugation at 10,000 rpm for 10 mins. One mg of the supernatant was pre-cleared with equilibrated protein G agarose beads for 2 hrs at 4 °C on a rotatory platform. Precleared lysate was then incubated with 2–5 µg of primary antibody overnight at 4 °C on a rotator. The lysate-antibody complex was then incubated with 50 µl of equilibrated protein G agarose beads (50/50 slurry) for 4 hrs at 4 °C. The beads were washed thrice with the lysis buffer at 4 °C for 5 mins each and then 2X protein loading dye was added to it and boiled for 10 mins; loaded on SDS-PAGE followed by immunoblotting.

### Immunocytochemistry

Cells were grown on coverslips coated with poly-L-lysine. Following treatments, cells were washed with PBS twice for 5 mins each and fixed with 3.7% formaldehyde in 10% FBS containing media for 15 mins and washed twice with PBS for 5 mins each. Cells were then permeabilized with 0.2% Triton-X-100 in PBS for 10 mins followed by two washes with PBS for 5 mins each. Cover slips were then kept in 1% BSA with respective primary antibodies at 4 °C overnight, followed by washing thrice for 5 mins and incubation in respective secondary antibodies (with Hoechst 1:1000) for 30 mins. The cover slips were then washed thrice with PBS for 5 mins each, mounted and sealed on slides. Images were captured with confocal microscope Olympus FV 1000.

### Fluorescence Activated Cell Sorting (FACS)

Cells were plated in 60 mm dishes and synchronised by double thymidine block. Cells were harvest at various time points by trypsinisation, collected by centrifugation at 200 g for 3 mins, followed by washing with PBS. They were fixed with 100% ice cold ethanol and kept at 4 °C overnight. Next day, they were washed with PBS and treated with 10 μg/ml RNase and 50 μg/ml of PI for 1 hr. Stained cells were analysed using BD flow cytometer.

### Quantitative RT-PCR (qRT-PCR)

Total RNA was isolated using TRI reagent (#T9424). cDNA was synthesized from 1 μg of RNA using Verso cDNA kit (#AB1453A, Thermo Fisher Scientific). Quantitative RT-PCR analysis was performed in 20 μl volume using 1X SYBR Green Master Mix (Applied Biosystems, USA). 18s rRNA was used as internal control for normalization. The normalized values were expressed as relative quantity of gene specific mRNA. Following primers were used for qPCR analysis of cyclinD1 gene: forward, 5′-CAGAGGCGGATGAGAACAAG-3′ and reverse, 5′-GAGGGTGGGTTGGAAATGAA-3′.

### Statistical analysis

Band intensities of western blots (n = 2–5) were quantified using ImageJ software. Statistical analysis was carried out from the quantified data and expressed in terms of Mean ± SEM and P value was calculated from one-way ANOVA. Significance was considered as follows for *P < 0.05, for **P < 0.01 and ***for P < 0.001.

## Electronic supplementary material

Supplementary Information
