# Author Correction: GSK3β and ERK regulate the expression of 78 kDa SG2NA and ectopic modulation of its level affects phases of cell cycle

**DOI:** 10.1038/s41598-018-37076-7

**Published:** 2018-12-17

**Authors:** Shweta Pandey, Indrani Talukdar, Buddhi P. Jain, Goutam K. Tanti, Shyamal K. Goswami

**Affiliations:** 1 School of Life Sciences, Jawaharlal Nehru University, 110067 New Delhi, India; 2 Department of Zoology, School of Life Sciences, Mahatma Gandhi Central University, Motihari, 845401 Bihar India; 3 Department of Neurology, School of Medicine, Technical University of Munich, Munich, Germany

Correction to: *Scientific Reports* 10.1038/s41598-017-08085-9, published online 08 August 2017

Goutam K Tanti was omitted from the author list in the original version of this Article. This has been corrected in the PDF and HTML versions of the Article, and in the accompanying Supplementary Information file.

The Author Contributions section now reads:

“S.K.G., I.T. and S.P. planned the experiments. S.P., I.T. and B.P.J. performed the experiments and analysed the data. G. K. T. did the preliminary experiments and generated data that is not included. S.P. and S.K.G. wrote the manuscript.”

