# Supplementary material for: GSK3β and ERK regulate the expression of 78 kDa SG2NA and ectopic modulation of its level affects phases of cell cycle
Source: Sci Rep. 2017 Aug 8;7:7555. doi: 10.1038/s41598-017-08085-9 (PMC5548716; doi:10.1038/s41598-017-08085-9)

GSK3β and ERK regulate the expression of 78kDa SG2NA and ectopic modulation of its level affects phases of cell cycle

Shweta Pandey, Indrani Talukdar, Buddhi P Jain, Goutam K Tanti, and Shyamal K. Goswami*

Supplementary Information File.

Supplementary Figure S1: Full length blots for all immunoblots.

Figure 1B

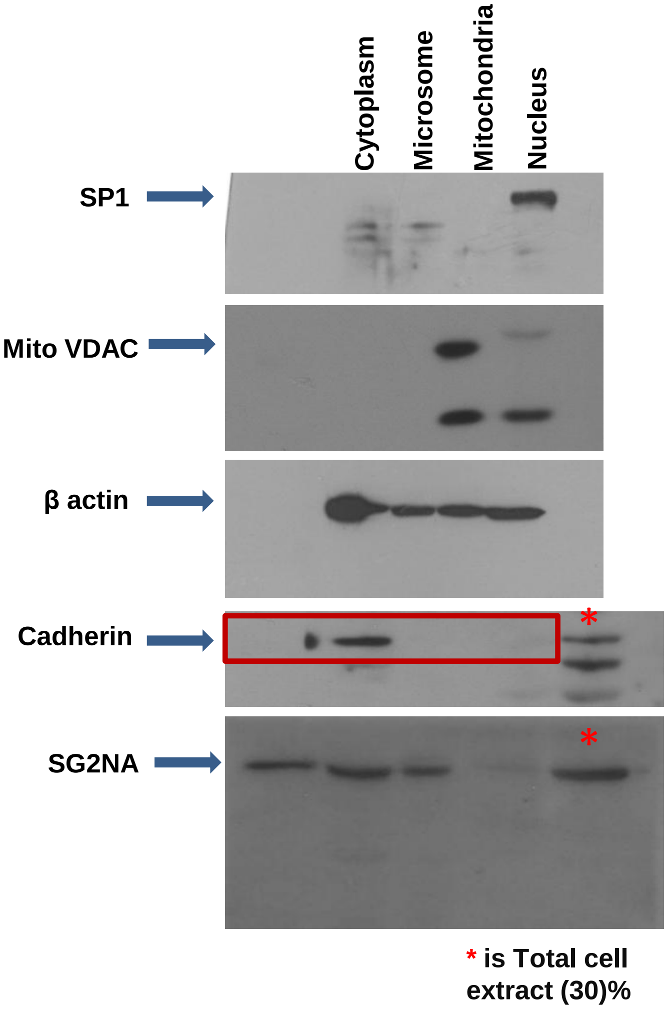

Figure 2B

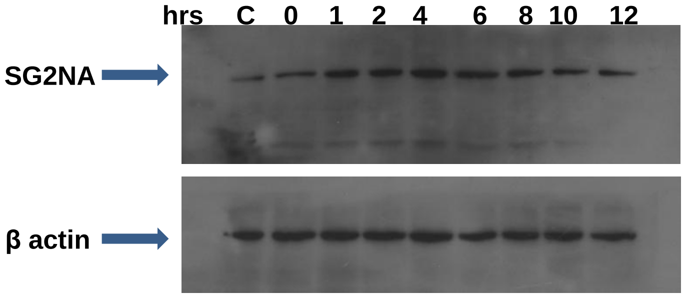

Figure 3B

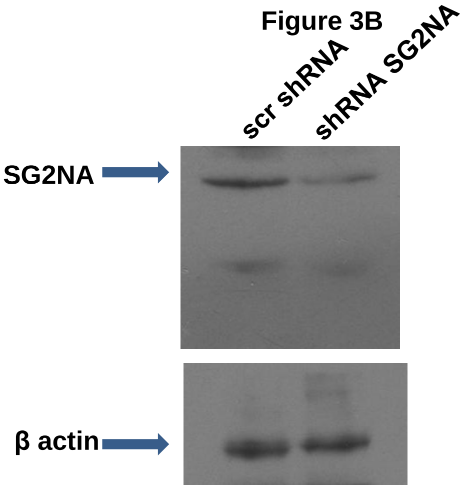

Figure 3A

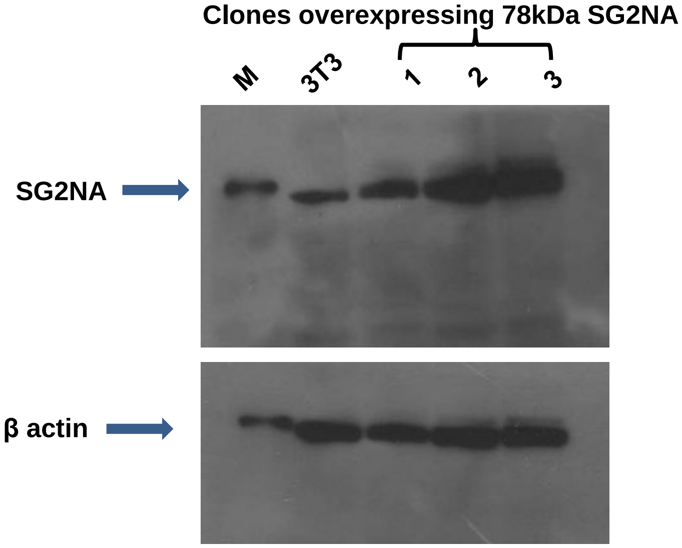

Figure 4A

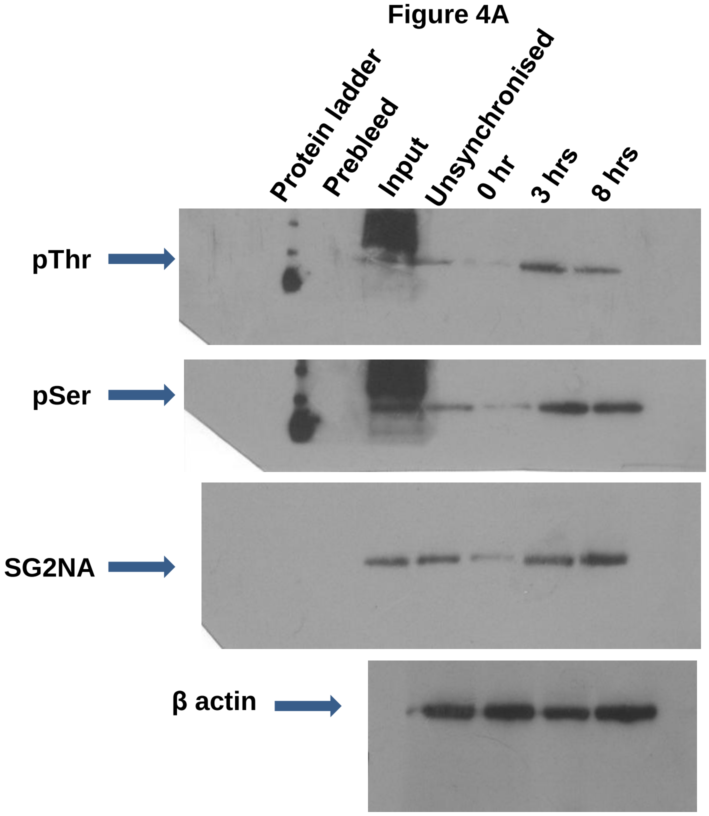

Figure 4C

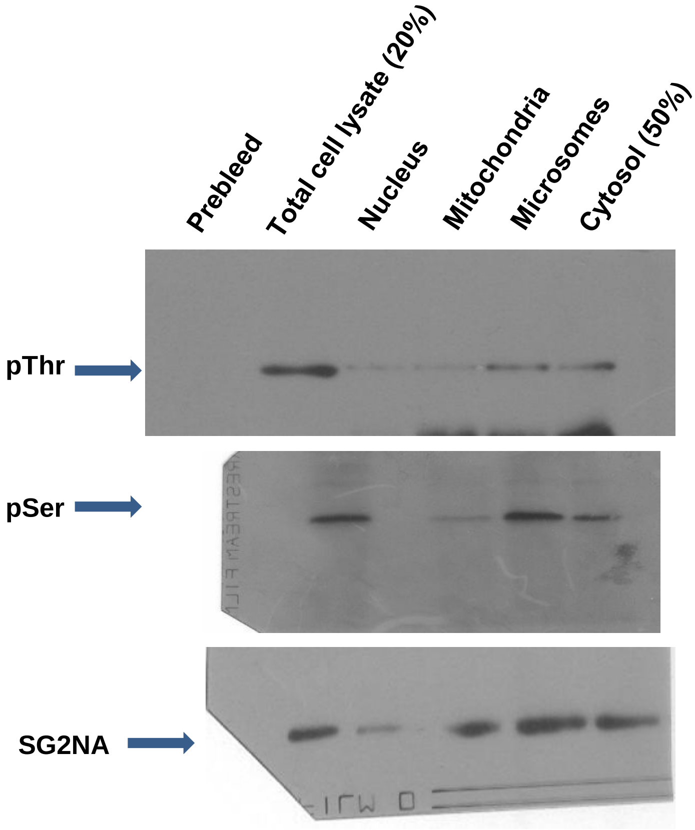

Figure 5A

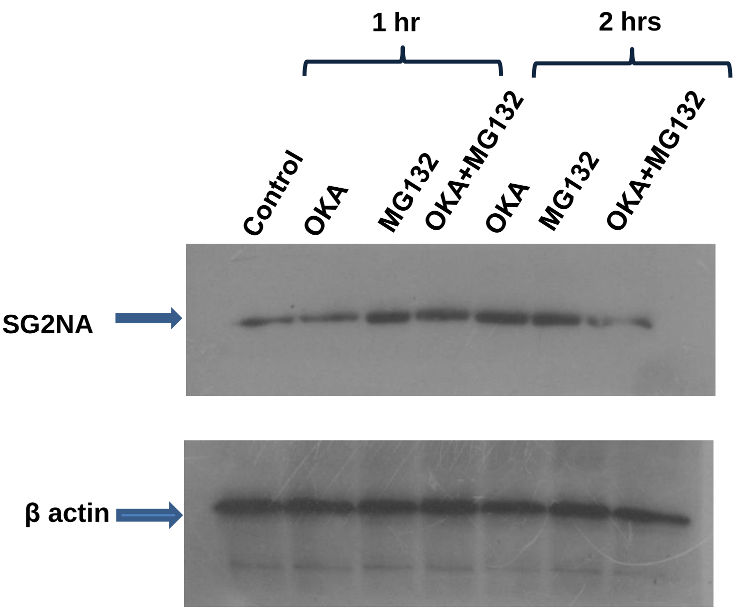

Figure 5C

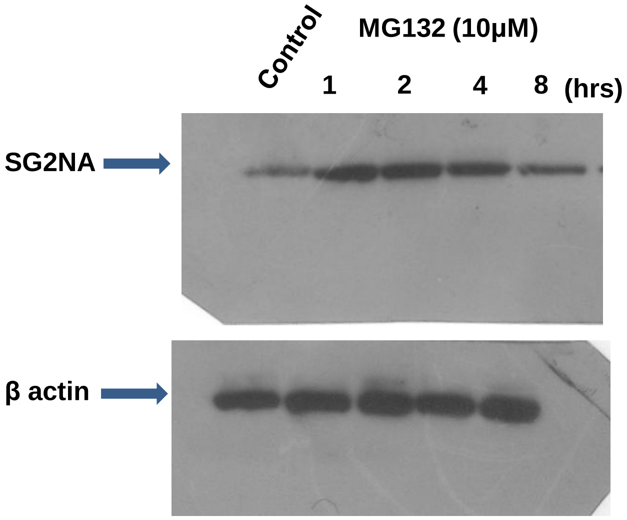

Figure 5E

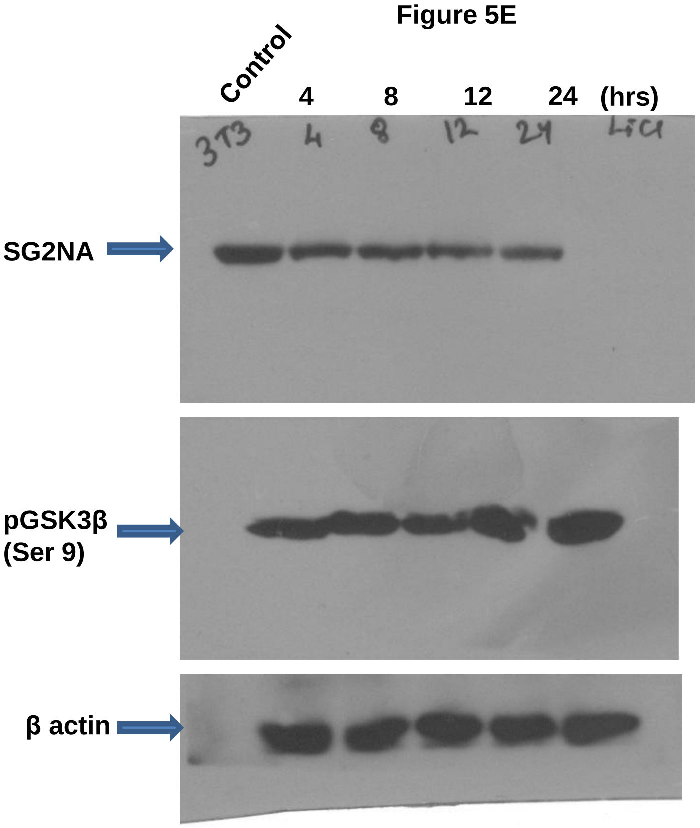

Figure 5H

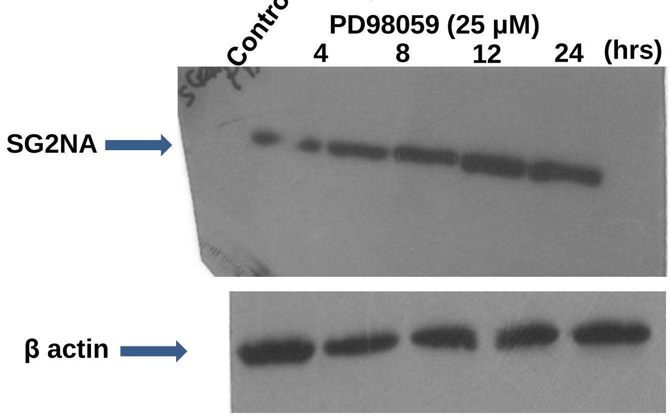

Figure 5K

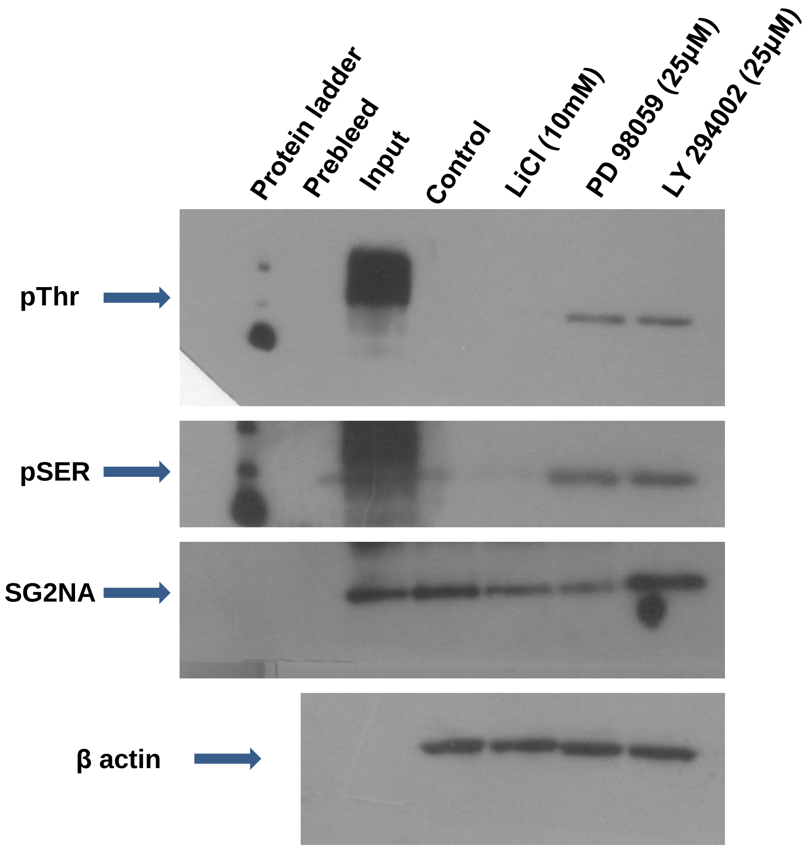

Figure 6A

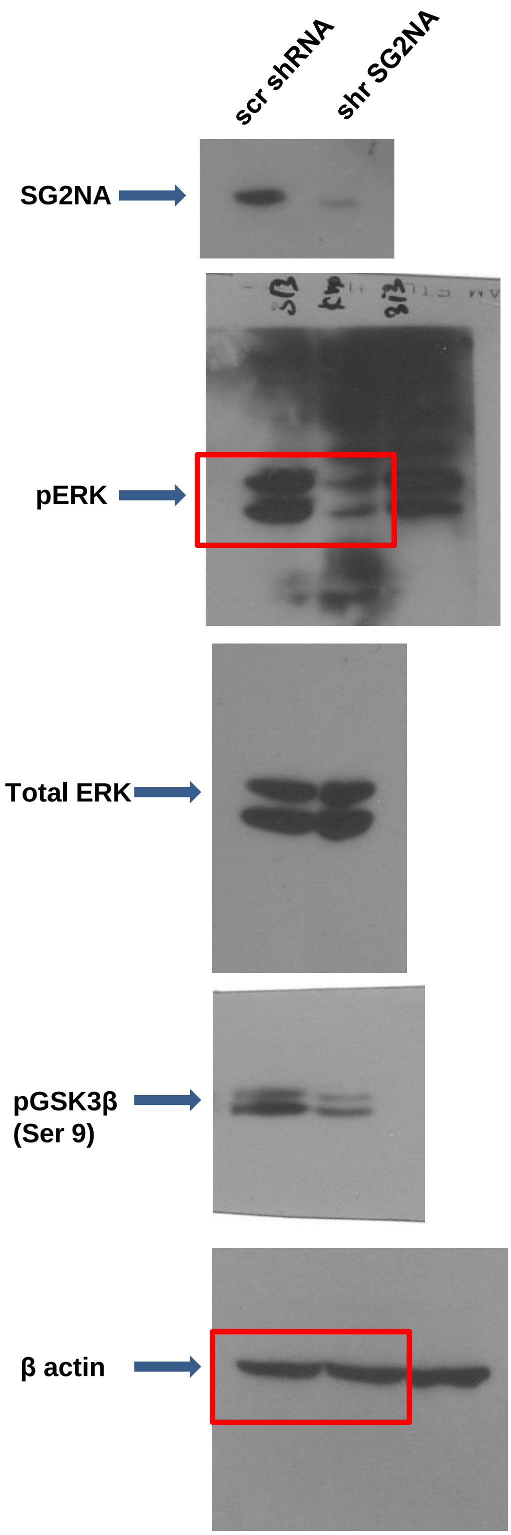

Figure 7A

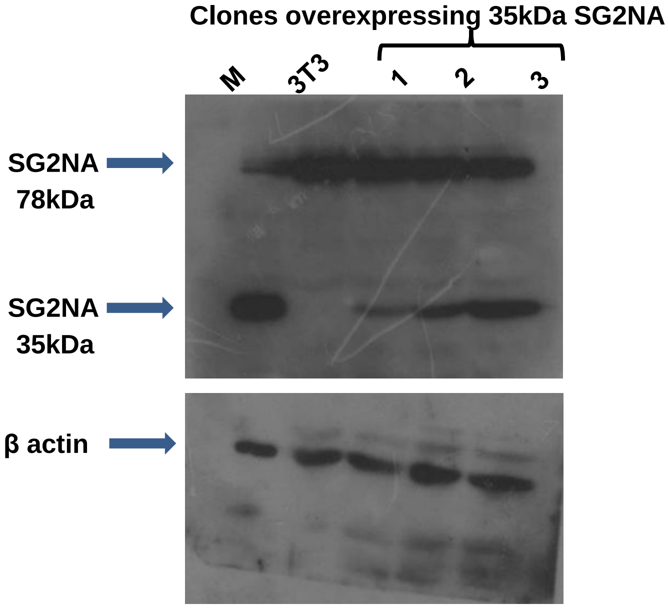

Supplement: Supplementary file 1 — Supplementary Information [file 41598_2017_8085_MOESM1_ESM.pdf]
